# Bacillus Calmette Guérin (BCG) vaccination use in the fight against COVID-19 – what’s old is new again?

**DOI:** 10.2217/fon-2020-0381

**Published:** 2020-05-14

**Authors:** Ellen O'Connor, Jiasian Teh, Ashish M Kamat, Nathan Lawrentschuk

**Affiliations:** ^1^Department of Surgery, University of Melbourne, Austin Hospital, Heidelberg, Melbourne, Victoria, Australia; ^2^Division of Cancer Surgery, Peter MacCallum Cancer Centre, Melbourne, Victoria, Australia; ^3^Department of Urology, Division of Surgery, University of Texas MD Anderson Cancer Center, Houston, TX 77030, USA; ^4^Department of Urology, The Royal Melbourne Hospital, Melbourne, Victoria, Australia

**Keywords:** BCG vaccine, clinical trials, coronavirus, COVID-19, immunotherapy, mycobacterium bovis

Bacillus Calmette Guérin (BCG) is a vaccine derived from the live attenuated strain of *Mycobacterium bovis* and used widely as a vaccination against tuberculosis in high-risk regions. The WHO recommend neonatal BCG vaccination in countries with high incidence of tuberculosis, with BCG being one of the safest and most widely distributed vaccines worldwide [1]. BCG is well known for its ability to induce a heterologous immunomodulatory effect on nonrelated conditions, a mechanism which is well understood and documented in the infectious disease literature. Most successfully, BCG is the most effective immunotherapy in oncology to date; it is used for treatment of nonmuscle invasive bladder cancer, being standard of care to achieve reduction in tumor progression and recurrence [2]. Global shortages of BCG in 2016 and again in 2019, driven by both increased demand and manufacturing constraints, have significantly impacted supply chains and resulted in change in practice in management of bladder cancer, with dose reduction and limitations in many countries. This treatment has been affected in recent years by global shortages of the agent. The COVID-19 pandemic has prompted urgent need for novel vaccination or means of reducing disease morbidity and mortality in the global community. Promising new trials aim to ascertain whether this commonly used vaccine has a role in the fight against COVID-19.

Since introduction of the BCG vaccine in 1921, an increasing body of evidence has demonstrated its ability to exert a range of nonspecific immunological effects beneficial for a range of other conditions. In epidemiological studies, neonatal BCG vaccination is associated with reduction in all-cause child mortality by 30% (0.70; CI 95%, 0.49–1.01), widely thought to be related to reduction in rates of neonatal sepsis and pneumonia [3]. BCG has the ability to train the innate immune system to generate an immune memory against secondary infections, a process also termed trained immunity [4]. This immune response has been shown to last up to 1 year following vaccination [5]. In mouse models, BCG was found to induce a trained immune response to avian influenza A (H7N9), however it was not associated with a clinical difference in survival, clinical scores or pulmonary inflammation [6]. *In vivo* studies have looked at effects of BCG vaccination on human monocytes following infection with yellow fever virus. Arts *et al.* successfully demonstrated epigenetic reprogramming of the innate immune system and reduction in yellow fever viremia [7]. The ability for BCG vaccination to induce a trained immune response to nonrelated pathogens raises the exciting possibility that it may have a role in protecting against the COVID-19 virus.

In several preprint manuscripts released online, authors have conducted epidemiological analyses of COVID-19 incidence in relation to nation-based BCG vaccination policies [8]. These studies observed a higher COVID-19 related morbidity and mortality in those countries which do not have a current or recent, universal BCG vaccination policy, suggesting that BCG vaccination may be a protecting factor. However, as with any observational epidemiological study, we caution that data such as these should be interpreted as hypothesis generating only. Given the widespread inconsistencies in collecting data relating to COVID-19 between countries, consideration of the stage of the COVID-19 pandemic in each country, differences in testing rates, isolation policies, national disease burden and demographics all must to taken into consideration. The WHO recently released a scientific brief cautioning against indiscriminate use of BCG in COVID-19 until appropriate evidence from ongoing studies becomes available [9].

Encouragingly, three new clinical trials have commenced recruitment aiming to test the hypothesis that BCG vaccination may be protective against COVID-19 in healthcare workers. The clinical trials, based in Australia (‘BRAVE’; PI Curtis), USA (‘BADAS’; PI: Kamat, Dinardo) and The Netherlands (PI: Netea), plan to randomize cumulatively greater than 6,000 healthcare workers to BCG vaccination versus a placebo agent. The primary outcome measures in each study differ; the Australian and American groups looking to evaluate the incidence of COVID-19 and severity of symptoms from disease and the Dutch group primarily looking at healthcare worker absenteeism [10–12]. A fourth, observational case-control, study based in Egypt has commenced recruitment examining COVID-19 positive patients comparing severity of disease in those who are tested positive for past BCG exposure or immunization to those who are tested negative [13].

With regards to patients receiving intravesical BCG therapy for bladder cancer, the potential effects of this treatment on COVID-19 infection is unclear. Mechanism of action following vaccination and topical intravesical administration differs significantly [14,15]. A pilot study has, however, demonstrated increased cytokine response in *ex vivo* monocytes of BCG-treated bladder cancer patients suggesting that intravesical administration of BCG may have the ability to induce a state of trained immunity to some degree [16]. Lastly, concerning the global shortage of BCG supply already affecting BCG treatment for patients with bladder cancer, careful consideration of appropriate usage of BCG is necessary. One vial of BCG used for bladder cancer can vaccinate up to 500 healthcare workers. It is encouraging that patient advocacy groups have come out with support for such trials [8]; it is up to us to ensure appropriate stewardship with regards to prevention of excessive depletion of already limited supplies.

## Conclusion

Undoubtedly the heterologous immune effects of BCG provide a promising avenue for investigation in relation to the COVID-19 pandemic. As cautioned by the WHO, appropriate evidence must be established prior to more widespread use for BCG for this purpose. Three new clinical trials aim to ascertain the protective role of BCG vaccination against COVID-19 virus, a possibility that would have global beneficial implications amidst the current pandemic.
